# The clinical effects of school sandplay group therapy on general children with a focus on Korea Child & Youth Personality Test

**DOI:** 10.1186/s40359-020-0378-9

**Published:** 2020-01-31

**Authors:** Hyeon Jeong Kwak, Un Kyoung Ahn, Myung Ho Lim

**Affiliations:** 1 Department of Psychology, Graduate School, Dankook University, Cheonan, South Korea; 2 Department of Psychology & Psychotherapy, College of Health Science; Environmental Health Center, Dankook Medical Hospital, Dankook University, 119 Dandae-ro, Dongnam-gu, Cheonan, 31116 South Korea

**Keywords:** School Sandplay group counseling, General children, The Korea Child & Youth Personality Test

## Abstract

**Background:**

This study intended to examine the comprehensive clinical effects of school sandplay group counseling on the emotions and behaviors of children for the first time in Korea.

**Methods:**

To this objective, 10 sessions of in-school sandplay group counseling were administered to 113 fourth- to sixth-graders in an elementary school located in Cheonan city for 12 weeks from March to July 2015. Each small group consisted of 10 to 16 children and the entire 12 sessions were composed of a baseline test, 10 therapy sessions, and a post-test and evaluation session. The study subjects consisted of 56 boys (49.6%) and 57 girls (50.4%). As the evaluation instruments, an epidemiologic questionnaire and the Korea Child & Youth Personality Test were used during the baseline phase and after the termination of the counseling.

**Results:**

The comparison of the scores according to the KCYP clinical scales and detailed evaluation scales before and after the 12-week counseling showed an increase in the self-esteem and a significant decline in depression in the elementary students after the counseling.

**Conclusion:**

It is deemed that school sandplay group counseling can help elementary school students to solve emotional problems and improve their self-esteem.

## Introduction

In the 1980s, while studying Lowenfeld’s Sandplay World Technique, Domenico developed a unique idea based on its communication function [1]. This was a different approach from Kalff’s individual sandplay therapy. Boik and Goodwin [2]. found that sandplay counseling for diverse clients can promote healing and growth as it does for individuals. Since then, clinicians have developed sandplay group counseling, widening the range of clients to adults and families [3]. Wang [4] noted that group counseling is more suitable than individual counseling for adolescents with problem behaviors and children in the stage of psychosocial development.

Children participating in group counseling interact with their peers honestly and experience emotional intimacy with others. Also, group counseling using sandplay is valuable as a school counseling tool. Sandplay, when used in school settings, is effective in releasing the blocked flow of energy inside children and activating their potential self-healing ability [5]. Dale [6] reported that sandplay therapy in school settings can be helpful to school violence victims as well as children who have difficulty participating in learning due to depression and suicidal ideation, severe conduct problems, and behavioral problems. Campbell [5] insisted that sandplay therapy in school improves interpersonal relationships and concentration of the children who have difficulty in communication. Sandplay therapy has also been reported to improve concentration and total kinesthetic involvement and alleviate problem behaviors in children with inattention. School group counseling is an important therapeutic intervention for emotional problems and behavioral behavior of school-age children.

This is because a school is the largest organization that can provide mental health services to children and adolescents and approach them in a flexible and effective way [7]. Sandplay therapy does not require a high number of sessions and has therapeutic effects even within a short period of time [5]. Therefore, school sandplay group counseling can meet the needs of school counselors who need to take care of many children in a short period of time [8]. Campbell and her colleagues found that most children who participated in eight to ten sessions of sandplay counseling in school enter a problem-solving stage within the period [5]. School teachers also reported significant changes in the children’s emotional state and their ability to participate in school life after the eighth session [5, 9].

This study uses the term “school sandplay group counseling,” which implies the condition that can be briefly expressed as “in school, with teacher, as a class.” “In school” refers to sandplay counseling conducted either at school or at a counseling agency entrusted by the local office of education. “With teacher” refers to an organic partnership with teachers or school counselors. Lastly, “as a class” suggests that the counseling be conducted for at least eight sessions as part of the school curriculum [10, 11].

In Korea, most studies on sandplay therapy have focused on individual cases, and those on group counseling have primarily presented cases of small groups of less than 20 clients that were mainly performed outside the school [10]. This study is Korea’s first research on sandplay therapy conducted in groups in school settings. The purpose of this study is to investigate the comprehensive clinical effects of school sandplay group counseling for a large number of students with respect to their emotions and behaviors.

## Methods

### Participants

Of the 303 children who had initially participated in school sandplay group counseling, 19 children were excluded. Fifteen children were excluded as they did not take the baseline and post-tests. Also excluded were four children who failed to acquire a significant level of a T-score (a score of 65) in response to the validity scales (response reliability, unusual reaction, exaggeration, and defensiveness) of the baseline and post KCYP tests. As a result, a total of 284 children were selected as the final study subjects, which consisted of 141 boys (49.6%) and 143 girls (50.4%). The average age of the study subjects was 10.94 ± 0.45 years and the children were in the fourth to sixth grades in elementary school (Table 1).

**Table 1:** The epidemiological characteristics of subjects

Variables	Subjects (*N* = 284)
	Mean(S.D.)
Age*	10.94 ± .45
Sex	N(%)
Male	141 (49.6%)
Female	143 (50.4%)

### Procedure

This study was conducted for three semesters from March 2015 to July 2016 and for one semester from March to July 2019. The school sandplay group counseling was conducted in class, for 40 min per session. A total of 12 sessions consisted of a baseline test, 10 therapy sessions, a post-test, etc. The entire class, divided into small groups of three to six, participated in the group counseling. The counseling was provided by a lead counselor who plans and oversees all of the counseling activities and three to six assistant counselors, who comprised certified sandplay therapy specialists or higher-level specialists.

The session process and program of the school sandplay group counseling were modified and supplemented to suit the characteristics of the school environment, based on the communication sand tray of Boik and Goodwin [2]. The session process and program were also reconstructed with the application of Kalff’s stages of ego development [12].

Based on Kalff’s Sandplay therapy, students were provided with individual sand tray 72 cm wide by 57 cm high and 7 cm deep. Principally, the children were required to maintain silence while creating their sand trays for better concentration. Children had a time to introduce their Sand Work to each other and to sympathize in silence and listened to and support their friend’s story telling of Sand Work. Also, Counselors recorded the interactions of communication and empathy, and observed the group dynamics and make therapeutic interventions. After the group counseling session, the figurines and miniatures in the trays were removed by the counselors, rather than by the children themselves, as part of an effort to provide the subjects a safe and sheltered space [21]. The session process, program and directives of the in-school sandplay group counseling are shown in [Table 2 and Table 3].

**Table 2:** Progress of sand play group counseling and contents of program

Step	Progress of sand play group counseling	Time (minute)
Step 1 Intro & promise	Introduction to sand play group counseling. (Only to talk about “promise together” for group counseling every time and talk aloud) Do not touch your friend’s work. Listen to your friend’s work description positively. Supports and speaks affirmative. (Do not swear)	5
	Close your eyes, touch the sand, use the touch, and get in with the rising emotions. (The leader counselor will guide the subject of the day to a quiet voice)	
Step 2 Create	After touching the sand a lot and making your own work quiet. (A client who wants to decorate freely and does not want to decorate is sitting quietly, touching the sand, or lying down)	15
Step 3 Share	Introduce your work to each group and share your feelings with your friends.	15
Step 4 To class	We promise to keep the story of the meeting during the consultation time as a secret between the members of the group. (Not being late, not being absent, not penalized)	5
Step 5 Clean up & record	After leaving the client, the counselor observes the client’s activities, records the work story, takes pictures, cleans up the work, and holds a counselor meeting with the school counselor.	40

**Table 3:** Directives of school sand play group counseling

Directives	Session	Contents of Directive words
Departure	1	Imagine departure of the journey of the heart. Express the feeling freely while touching the sand.
Search (Self-emotional contact and expression)	2	Close your eyes. Touch the sand. Feel the basic emotions of sadness, joy, pain, happiness, and express them in a sand tray.
	3	Remind yourself of the events and situations you’ve been through and freely express your inner feelings.
Conflict and struggle	4	Feel negative emotions such as conflict and struggle and express them freely
	5	Imagine and express freely the heroes who face difficulties, hardships and adversity
Relationship (Family, friend, school)	6	Express feelings as you think about your family
	7	Express your feelings in the sand tray while thinking about your friends and school.
Self understanding and acceptance	8	Express yourself in the sand tray. (Including past, present, future me, negative me, positive me)
	9	Recall your sand work you’ve made up until now. Express the center of my heart and treasure.
New birth	10	Imagine and express yourself in the new me, the future.

Pre/Post test is carried out separately.

### Measure

The Korea Child & Youth Personality Test (KCYP) is a test that is developed to examine the personality traits of the age group of third-graders in elementary school to second-year students in high school. The test consists of 237 questions and includes the US Personality Inventory for Children, as well as a test on game and Internet addiction tendencies [13]. The self-report questionnaire for children comprises questions that can be answered with either “True” or “False,” while the questionnaire for parents is composed of questions with four-point scale response options (True/ Some true/ Some false/ False). Validity scales consist of response reliability, unusual reaction, exaggeration, and defensiveness. The clinical scales include ego strength, psychotic symptoms (reality distortion, hallucination), somatization (psychogenic disorder, tension/anxiety, hypochondria), emotional discomfort (phobia, depression, sleep problems), social withdrawal (introversion, isolation), ADHD (inattention, hyperactivity), delinquency (oppositionality, conduct problem), social relationships (isolation from peers, conflict with peers), family relationships (conflict with parents, conflict among family members), learning disability, indulgence in media, etc. The test-retest reliability of this test in terms of the response tendency is .65–.85, and the test-retest reliability in the personality trait area is .74–.89 [21]. The internal consistency (Cronbach ‘s α) of the test in this study was .86.

### Data analysis

To compare the changes of general child group between before and after the sandplay therapy, paired t test was performed. And chi square test was performed for sex, and t- test was performed for age.

### Ethics statement

Ethical statement: All of the subjects were provided with a comprehensive explanation of the study and its objectives, and oral and written consents were obtained from each of the subjects and their parents. They were also informed that they can withdraw from the study at any time during the research period according to their own will. The patient provided written informed consent. This study was approved by the Dankook Hospital Ethics Committee (2019–05-003).

## Results

In a paired t-test, statistically significant differences in emotional and behavioral factors were observed after the completion of sandplay counseling compared to the baseline.

The school sandplay group counseling conducted in this study had a positive effect on the subjects’ emotional problems such as reality distortion, hallucination, tension/anxiety, hypochondria, phobia, sleep problems, introversion, etc. among the participating children (Table 4).

**Table 4:** Changes in KCYP after 10 weeks of sandplay therapy

Scale	Before sandpaly therapy (*n* = 284) M ± SD	After 10 weeks of sandplay therapy (*n* = 284) M ± SD	t	*p*-value
Reliability				
Response reliability	47.98 ± 9.67	45.65 ± 9.52	4.028	<.001
Infrequency	48.02 ± 9.28	46.81 ± 9.47	2.224	.027
Exaggeration	50.45 ± 9.81	50.9 ± 10.26	−.906	.366
Defense	50.14 ± 10.33	49.79 ± 9.44	.657	.512
Ego strength	54.32 ± 10.26	55.87 ± 10.44	−3.781	<.001
Psychosis				
Reality distortion	50.58 ± 9.99	49.21 ± 9.83	2.693	.008
Hallucination	50.85 ± 10.37	48.49 ± 8.80	4.332	<.001
Somatization				
Psychosomatic	49.99 ± 9.78	47.99 ± 9.60	1.841	.067
Tension/Anxiety	49.46 ± 9.56	47.48 ± 9.42	4.145	<.001
Hypochondriacs	49.60 ± 10.47	47.79 ± 9.42	3.308	.001
Emotional distractibility				
Phobia	48.54 ± 10.16	46.23 ± 10.16	4.295	<.001
Depression	48.62 ± 9.68	46.23 ± 10.07	.789	.431
Sleep problems	50.08 ± 11.21	48.38 ± 10.16	3.325	.001
Withdrawal				
Introversion	48.31 ± 10.17	46.75 ± 9.88	2.954	.003
Isolated feeling	49.32 ± 8.70	48.50 ± 7.98	1.504	.134
ADHD				
Inattention	48.71 ± 9.41	46.86 ± 9.03	3.304	.001
Hyperactivity	48.88 ± 9.34	48.07 ± 9.42	1.599	.111
Delinquency				
Oppositionality	46.54 ± 17.49	44.77 ± 17.87	2.015	.045
Conduct problem	48.59 ± 19.36	45.63 ± 19.58	2.700	.007
Social relation				
Peer isolated feeling	48.39 ± 18.06	47.06 ± 18.92	1.483	.139
Peer conflict	44.78 ± 16.82	45.43 ± 16.42	−.813	.417
Family relation				
Parent conflict	47.38 ± 16.43	43.59 ± 15.91	4.276	<.001
family discord	45.46 ± 19.65	45.90 ± 19.72	−.530	.596
Learning difficulty	48.11 ± 9.58	47.56 ± 10.01	.916	.361
Media Indulgence	49.54 ± 9.42	49.11 ± 9.34	.846	.398

Also, the counseling resulted in the alleviation of their cognitive and behavioral problems including inattention and delinquency (oppositionality, conduct problem), conflict with their parents as well as an increase in ego strength (Table 4).

## Discussion

Christine [10, 11] reported that children who had experienced trauma after the September 11 attacks showed the alleviation of negative feelings and emotions or the decrease in their intensity. Wang [4] presented a case of sandplay group counseling for children with conduct problems. In this case, significant differences in internalization problems such as somatization, introversion, and depression were observed before and after the counseling.

Studies carried out in Korea have also reported among subjects reduced internalizing internalizing problems such as introversion, somatic symptoms, depression, and anxiety after administering sandplay group counseling [12]. In the study by You [13], children’s anxiety was reduced and self-resilience was improved after sandplay group counseling. The study by Kim [14] also reported the clinical effect of sandplay group counseling on children’s self-resilience. Park [15] noted that sandplay group counseling has a positive effect on the relief of anxiety and ego strength in children. In the study by Mun [15], the children who participated in sandplay group counseling experienced a decreased level of depression and increased self-esteem. In this study, however, there was no significant difference in depression, which is an emotional disorder of children, before and after the counseling.

Given that children tend to express their feelings of depression, anxiety, and fear through somatic symptoms (e.g. stomachache and headache), from the positive effect of the counseling on tension/anxiety, hypochondria, and phobia in the study subjects, it was presumed that sandplay group counseling is effective in alleviating emotional problems similar to depression. This is consistent with the findings of the precedent studies [12, 16] that reported a decreased level of depression in children who participated in sandplay group counseling. In addition, Ahn [14] reported that sandplay group counseling for 20 high school students with difficulty adapting to school life had a positive clinical effect on the adolescents who had complained of depression accompanied by somatic symptoms. The study results suggest that sandplay group counseling is clinically effective in lowering the level of depression not only in children but also in adolescents.

Sandplay counseling positively affects the mitigation of problem behaviors in children [17]. Wang [4] reported that sandplay group counseling for children with conduct problems led to significant differences in externalizing problems such as noncompliance to parental expectations, violation of rules, and aggression. Among Korean researchers, Lee [12] found that sandplay group counseling for children decreased their delinquency and aggression and improved their peer relationships. In the study by Byeon [18], sandplay counseling alleviated problem behaviors in children with inattention. While the study by Park [19] reported that group sandplay counseling on school violence victims had a positive effect on school adjustment. In the study by Han [20], sandplay counseling for children with externalizing behavioral problems resulted in decrease in their aggression and negative peer interactions.

On the other hand, this study observed statistically significant differences in reality distortion, hallucinations, sleep problems, healthy family relationships, and conflict with parents, which affects children’s problem-solving ability, before and after the counseling.

This consists with the findings of Jeong’s study [24]^15^, suggesting that a healthy family relationship, which is closely related to children’s conflict with their parents, affects their ability to adapt to school life and solve problems. From this finding, it is considered that sandplay counseling also positively affected reality distortion. It is, therefore, deemed that the school sandplay group counseling had a significant effect on the school life adjustment and problem-solving ability of the children who participated in the study.

This study intended to investigate the comprehensive clinical effects on the emotions and behaviors of children who received school sandplay group counseling, a subject that has been studied for the first time in Korea. The results of this study established the clinical effects of in-school sandplay group counseling on children’s emotions with statistically significant differences in the KCYP Test after the counseling, compared to the baseline. The clinical effects on children’s emotional and behavioral/social symptoms such as ego strength, reality distortion, hallucination, tension/anxiety, hypochondria, phobia, sleep problems, introversion, inattention, oppositionality, conduct problems, parent conflict, etc. were measured. Such study results consist with the findings of the various precedent studies [4, 6, 11–16, 18–20] that reported the clinical effects of sandplay counseling on emotional symptoms or behavioral/social symptoms.

Nevertheless, there are some limitations to this study. First, as the KYCP Test is a self-report test, it has limitations in the identification of individuals’ pathological traits.

Second, as this study was conducted on the students living in a specific region, its results cannot be generalized and considered to reflect the characteristics of all students of the same age group. Third, this study only compared the pre- and post-conditions of the group that participated in the in-school sandplay group counseling.

While the counseling in this study was structured as a group counseling program involving the entire class, there is a need for follow-up studies that compare a subject group of children with emotional and behavioral problems with another general control group, in order to identify the advantages and disadvantages of school sandplay group counseling. Precedent Korean studies that have been published to date have presented cases of sandplay counseling for individuals or small groups of 3 to 16 [9]. However, there was some variation among the studies. For example, in some studies where group counseling was conducted, a model combining individual and group counseling was used [10, 13, 14].

In consideration of this account, the significance of this study lies in that it is Korea’s first study to conduct school sandplay group counseling for a large number of children (*n* = 284) and that it verified the comprehensive clinical effects of sandplay group counseling through this project. Based on the results of this study, it is expected that sandplay group counseling will serve as a useful means of intervention in school settings.

## Conclusion

This study administered in-school sandplay group counseling to fourth- to sixth-graders in elementary school for 12 weeks. As a result, it was verified that the counseling led to statistically significant differences in the emotional scales including reality distortion, hallucination, tension/anxiety, hypochondria, phobia, sleep problems, introversion, etc., and in the behavioral scales including inattention, conduct problem, etc., compared to the baseline value. A significant increase in ego strength and a decrease in conflict with parents was also observed. Based on the results of this study, it is assumed that school sandplay group counseling has clinical effects on the emotions and behaviors of the participating children.

## Data Availability

The datasets generated during and/or analyzed during the current study are available from the corresponding author on reasonable request.

## Abbreviations

KCYP: Korea Child & Youth Personality Test

## References

[1] Domenco GS (2002). Sandtray-worldplay: A psychotherapeutic and transformational sandplay technique for individuals, couples, families and groups. Winter 2002. Sandtray Netw J.

[2] Boik GL, Goodwin EA (2000). Sandplay therapy: A step by step Manual for psychotherapists of diverse orientations.

[3] Christian R (2019). Sandplay therapy: An overview of theory, applications and evidence base. Arts Psychother.

[4] Wang MH (2007). Effect of group sandtray therapy with preadolescents. J Spec Group Work.

[5] Goss S, Campbell MA (2004). Value of Sandplay as a Therapeutic Tool for School Guidance Counsellors. Australian J Guid Counsel.

[6] Dale A, William J, Sandplay LM (2000). A Constructivist Strategy for Assessment and Change. J Constr Psychol.

[7] US Department of Health and Human Services (2000). US Department of Education. Report of the Surgeon General's Conference on Children's Mental Health: A National Action Agenda.

[8] Drewes Athena A., Schaefer Charles E. (2010). School-Based Play Therapy.

[9] Allan J, Berry P (1987). Sandplay. Elem Sch Guid Couns.

[10] Kwak HJ, Ahn UK, Han GJ, Lim MH (2018). The Clinical Effect of School Sand Play Group Counseling on Child Emotion and Behavior. J Content Assoc.

[11] Christine JY, Sara MA, Violeta EM, Mio T (2015). The Use of Sandplay Therapy in Urban Elementary Schools as a Crisis Response to the World Trade Center Attacks. Psychol Res.

[12] Kalff D. Introduction to Sandplay Therapy. J Sandplay Therapy. 1991;1(1):9–15.

[13] Lim HC (2015). Korean Child & Youth Personality Test Interpretation.

[14] Ahn UK, Kwak HJ, Kim JW, Lim MH. The Clinical Effect of Sandplay Therapy on Adolescent Behavior and Emotion - Through Minesota Multiphasic Personality Inventory. J Content Assoc. 2017;17(12): 257–265. DOI. 10.5392/JKCA.2017.17.12.257.

[15] Jung YJ (2017). A study on the effect of family strength on school adjustment of elementary students and the mediating effect of problem solving skills. J Content Assoc.

